# Abnormal tau induces cognitive impairment through two different mechanisms: synaptic dysfunction and neuronal loss

**DOI:** 10.1038/srep20833

**Published:** 2016-02-18

**Authors:** J. Di, L. S. Cohen, C. P. Corbo, G. R. Phillips, A. El Idrissi, A. D. Alonso

**Affiliations:** 1 Department of Biology and Center for Developmental Neuroscience, College of Staten Island, City University of New York, Staten Island, NY 10314, USA; 2 Department of Biological Sciences, Wagner College, Staten Island, NY 10301, USA

## Abstract

The hyperphosphorylated microtubule-associated protein tau is present in several neurodegenerative diseases, although the causal relationship remains elusive. Few mouse models used to study Alzheimer-like dementia target tau phosphorylation. We created an inducible pseudophosphorylated tau (Pathological Human Tau, PH-Tau) mouse model to study the effect of conformationally modified tau *in vivo*. Leaky expression resulted in two levels of PH-Tau: low basal level and higher upon induction (4% and 14% of the endogenous tau, respectively). Unexpectedly, low PH-Tau resulted in significant cognitive deficits, decrease in the number of synapses (seen by EM in the CA1 region), reduction of synaptic proteins, and localization to the nucleus. Induction of PH-Tau triggered neuronal death (60% in CA3), astrocytosis, and loss of the processes in CA1. These findings suggest, that phosphorylated tau is sufficient to induce neurodegeneration and that two different mechanisms can induce cognitive impairment depending on the levels of PH-Tau expression.

Human tau has been implicated in the pathogenesis of several neurodegenerative diseases, including Alzheimer’s disease (AD), Fronto-Temporal Dementia, Chronic Traumatic Encephalopathy, Pick’s Disease, tangle-only dementia, and others, collectively known as tauopathies^1^,^2^,^3^. Although the mechanisms underlying the development of tauopathies are not fully understood, it has been shown that cognitive decline in neurodegenerative diseases correlates with deposits of hyperphosphorylated tau^4^. Tau hyperphosphorylation can lead to abnormal folding, fragmentation, aggregation and/or the development of deposits known as neurofibrillary tangles (NFTs)^5^.

Most AD models used today are generated based on mutations of amyloid-β (Aβ) or Presenilin-1 (PS-1), found in familial AD (fAD). These mutations account for only a small percentage of the cases of AD^6^,^7^. Models with both Aβ and PS-1 mutations exhibit amyloid deposition and show cognitive deficits as well as age dependent decline in spatial memory and long-term potentiation. Mouse models which overexpress forms of human tau have been generated and reproduce cognitive impairment^8^,^9^,^10^,^11^,^12^,^13^ and neuronal death^14^. In most of these models, the level of heterologous tau expression is very high compared to endogenous tau. Human tau has been expressed in various isoforms, without the mouse tau, or with mutations found in Fronto-Temporal Dementia with Parkinsonism linked to chromosome 17 (FTDP-17)^15^,^16^,^17^,^18^,^19^,^20^,^21^,^22^,^23^,^24^. These mice show neurodegeneration, but also manifest symptoms that are not commonly linked to AD. For example, expression of MAPT driven by the mouse prion promoter results in accumulation of tau in the spinal cord leading to motor deficiencies^15^,^22^. Recently, groups have used the Tet-Off system which utilizes a Ca^2+^/Calmodulin dependent protein kinase II (CamKII) promoter to drive expression in neurons and to control the timing of the tau protein expression using doxycycline to regulate the promoter^19^,^23^. In most of these transgenic models, the tau that is expressed becomes phosphorylated at common Ser/Thr residues, some of which are known to be pathogenic phosphorylation sites^25^,^26^,^27^,^28^.

The generation of mouse models that express tau phosphomimetics may be useful in the development of therapeutics for treatment of dementia caused by hyperphosphorylation of tau. Phosphomimetics introduce negative charges on tau similar to phosphorylation events which may change the protein conformation. A mouse model in which 10 different sites on tau were pseudohyperphosphorylated was generated but no indications of neurodegeneration or differences in learning or memory were detected^23^. We have demonstrated that tau pseudophosphorylated at Thr212, Thr231 and Ser262 mimics AD abnormal tau^29^ and that tau pseudophosphorylated at Ser 199, Thr212, Thr231 and Ser262 impairs learning and memory in Drosophila^30^. These sites are present early in AD pathology^25^,^26^, and they have very interesting implications. For example, Ser199 is found in CSF of AD patients and used as a marker^31^. Thr212 can be phosphorylated by the dual-specificity tyrosine(Y)-phosphorylation–regulated kinase 1A (DYRK1A), which is coded in chromosome 21. The amyloid precursor protein (APP) is also coded in chromosome 21; therefore, with the extra copy of chromosome 21, Down syndrome (DS) patients have three copies of DYRK1A and APP explaining why most individuals with DS show early-onset of AD^32^. Ryoo *et al.* (2008) generated transgenic mice over-expressing DYRK1A and these mice have increased tau phosphorylation at Thr212; this hyperphosphorylated tau did not promote microtubule assembly^33^. Thr212 is situated in a very basic domain of tau, so the impact of phosphorylation on this site is very strong^26^. With respect to Thr231 and Ser262, there is evidence that those sites are very important in tau binding to microtubules and that the combination of phosphorylation at these sites dramatically decreases tau microtubule–promoting activity^34^. More exciting even was to learn that by using human fibroblast from familial and sporadic AD patients and controls they were able to generate induced pluripotent stem cells. Upon differentiation into neurons they found there is an increase in phosphorylated tau at Thr231 in cells obtained from AD patients compared with those of the controls^35^. We showed that the toxic effect was stronger when the sites chosen for pseudophosphorylation were paired with the FTDP-17 mutation R406W^29^,^30^. Based on these different observations it appears that the specific sites that are phosphorylated or modified, rather than the number of sites, may be an important factor in tau toxicity.

To carefully probe the relationship between phosphorylation, expression level, neurodegeneration and cognition we generated a mouse model in which expression of tau with pseudophosphorylated sites at Ser199, Thr212, Thr231, and Ser262 as well as the R406W mutation (pathological human tau, PH-Tau, Fig. 1A) could be regulated. The expression of the PH-Tau is controlled by the Tet-Off system which can be regulated by the addition (suppressed) or removal (induced) of doxycycline to the food and/or water of these animals. By controlling the expression, we can mimic the sporadic form of AD in which hyperphosphorylation may be triggered by environmental factors, stress, traumatic brain injury, or another unknown cause.

We found that double transgenic mice in which PH-Tau is suppressed still expressed baseline levels of PH-Tau (~4% of total tau protein, PH-Tau_low_). At this low level, PH-Tau is detected as oligomers and its expression triggers early cognitive deficits which may be caused by loss of synapses in the hippocampus. These cognitive deficits appear to be more significant than in the mice in which expression of PH-Tau is induced (see below). To our knowledge, this is the first model where barely detectable levels of abnormal tau can cause dramatic effects. Upon induction, PH-Tau expression increases up to 14% of total tau protein and aggregates can be detected (PH-Tau_high_). PH-Tau expression is observed in the forebrain of the mice and expression results in cognitive decline (less than PH-Tau_low_ mice), significant neuronal loss, and astrocytosis. We believe this is the first evidence of two different mechanisms leading to cognitive decline that may be the result of varying levels of PH-Tau expression.

## Results

### Characterization of PH-Tau expression

**Figure 1:**
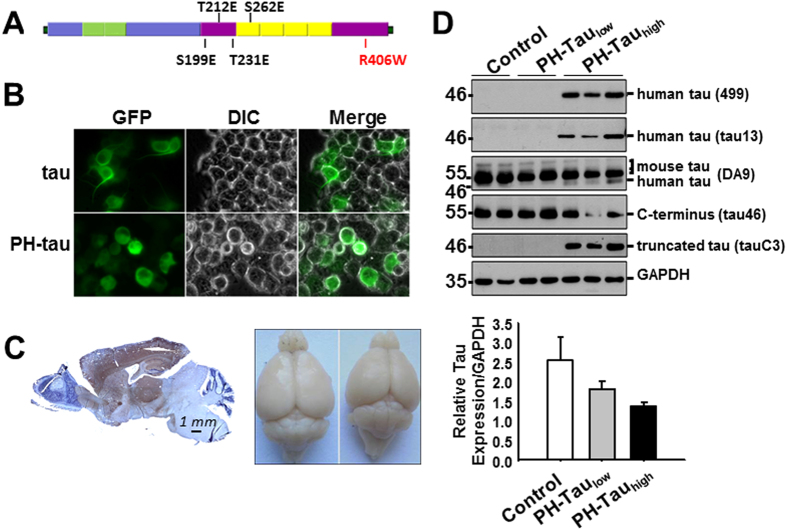
Generation of inducible transgenic mice expressing PH-Tau resulting in cognitive impairments in bigenic mice. (**A**) Illustration of full-length tau with pseudophosphorylation and mutation sites marked. (**B**) Live imaging of N2A cells transfected with plasmids expressing tau and PH-Tau. Neurite formation is observed in the presence of WT tau, but the cells become rounded when PH-Tau is expressed. (**C**) **Left:** Sagittal paraffin section of PH-Tau expressing mouse stained with human-Tau antibody Tau-13 (brown) and counterstained with hematoxyline (blue). **Right:** Brains of control (left) and PH-Tau_high_ (right) mice showing a loss of size when PH-Tau is expressed. (**D**) Tau proteins were examined in hippocampus homogenates. The graph at the right was determined by performing densitometry using the DA9 antibody.

Previously we have shown that when PH-tau was expressed in CHO cells it was aggregated in the cells, disrupted microtubules, and translocated in the nucleus^29^. When expressed in Drosophila, it induced mushroom body disruption and cognitive impairment^30^. To check the effect on a neuronal-like cell, we transfected tau and PH-Tau into N2A cells (Fig. 1B). Tau expression induced process formation whereas PH-Tau expression failed to induce neurites and translocated in the nucleus of N2A, as seen by a 143% increase in green fluorescence in the nuclear region in PH-Tau cells relative to WT.

We created transgenic mice carrying the PH-tau gene as an inducible transgene so that expression was driven by the forebrain-specific CaMKII promoter (inducer mice) and regulated by the addition of doxycycline to the diet. Bigenic mice were generated by mating the responder mice with the inducer mice and upon induction (removal of doxycycline from diet), transgenic tau expression was largely restricted to neurons of the forebrain with highest levels in the cortex, hippocampus, striatum, and amygdala (Fig. 1C, left). No expression was observed in the cerebellum and brain stem. Human-tau positive cells were not observed in the brains of non-transgenic littermates or single transgenic mice. We also observed that there was a reduction in the size of the mouse forebrain after induction of PH-Tau for 5 months (Fig. 1C, right).

To study the effect of PH-Tau on mature neurons, expression was induced in one year old mice for 3 to 12 months. PH-Tau was detected by western blot of brain homogenates using two different anti-human tau antibodies (tau13 and 499). A single band with an apparent molecular weight of 46 kDa in PH-Tau_high_ mice was detected. No human tau positive band seen in non-induced littermates under these conditions (Fig. 1D). The pan-tau antibody DA9 identified three endogenous mouse tau isoforms (50–60 kDa, Fig. 1C)^12^, as well as the 46 kDa band in PH-Tau_high_ mice. PH-Tau was recognized by tauC3 (antibody against truncated tau at Asp421) showing that PH-Tau is truncated at D421 (Fig. 1D), implicating caspase 3 activation in these transgenic mice^36^. Conversely, tau46 antibody (antibody against the C-terminus) did not recognize the truncated human tau but rather recognized the endogenous mouse tau isoforms (Fig. 1D)^37^. Interestingly, the amount of endogenous mouse tau decreased in PH-Tau_high_ mice compared to control mice (Fig. 1D, DA9 and tau46). The expression of PH-Tau relative to the total tau in the cell was judged by densitometry to be 14% in the PH-Tau_high_ mice.

### Low level PH-Tau results in oligomeric species

**Figure 2:**
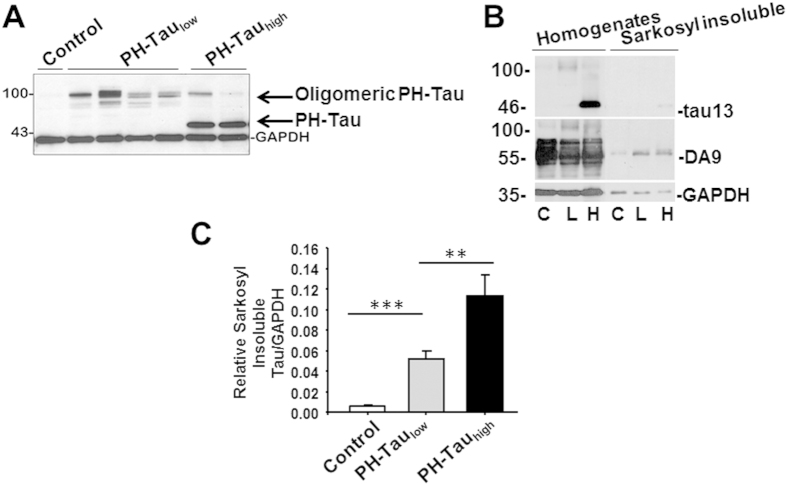
Tau protein characterization. (**A**) Hippocampal homogenates incubated at 37 °C for 1 h resulted in the appearance of a ~100 kDa species visualized with tau13 antibody in both bigenic mice. (**B**) Human and mouse tau proteins were measured in the sarkosyl fractionates of forebrain homogenates. C: Control, L: PH-Tau_low_, H: PH-Tau_high_, GAPDH as loading control. (**C**) Quantitation by densitometry showed sarkosyl insoluble tau increased significantly (***P < 0.001) in PH-Tau_low_ mice *vs* control, and further increased (**P < 0.01) in PH-Tau_high_ *vs* PH-Tau_low_ mice (n = 3 per group).

Results from another inducible mouse model indicated that the expression of tau was not completely suppressed by doxycycline treatment^20^. In classical Western blot with samples heat denatured for 5 minutes at 95 °C (Fig. 1D), the 46 kDa truncated PH-Tau was undetectable in mice on a continuous doxycycline diet. However, we modified the sample preparation to look for potential aggregate formation, which has been shown to occur upon heating for proteins interacting with membranes^38^. We speculated that the PH-Tau may have formed large aggregates upon heating and were unable to enter the gel under the conditions of Fig. 1D. The samples were therefore incubated at 37 °C for 1 h, rather than boiled, prior to electrophoresis. Under these conditions, oligomers similar to those previously described for tau^39^ were visualized at ~100 kDa with tau13 in both bigenic mice (Fig. 2A). The aggregates were also recognized by DA9, tauC3 and tau46 antibodies (data not shown). Densitometry was used to determine the amount of PH-tau in the oligomers of PH-Tau_low_ mice compared to the amount of PH-tau in PH-Tau_high_ mice. Using an antibody specific for human tau, the amount of oligomeric tau in PH-Tau_low_ mice was determined to be about 30% of PH-Tau in PH-Tau_high_ mice (Fig. 2A). Based on this and the amount of total tau in these mice (Fig. 1D) the level of tau in the PH-Tau_low_ mice was calculated to be ~4% of total tau. It is notable that the amount of tau oligomers decreased upon induction of PH-Tau expression.

### Insoluble Tau observed in bigenic mice

Sarkosyl-insoluble tau aggregates are a hallmark of AD biochemistry. Sarkosyl-insoluble tau was observed in homogenates of PH-Tau_high_ mice with human tau (tau13) and pan-tau (DA9) antibodies, whereas, in PH-Tau_low_ mice it was only observed with pan-tau antibodies (Fig. 2B). These results suggest that the PH-Tau_low_ mice with barely detectable PH-Tau levels induced sarkosyl-insoluble aggregates containing mouse tau. We have shown previously that with protein purified from human tissue, AD P-Tau can recruit normal tau to the aggregates^40^. The sarkosyl-insoluble tau increased significantly in PH-Tau_low_ mice compared to control 5.1% ± 0.5% (P < 0.001), and further increased in PH-Tau_high_ mice 11.3% ± 1.2% (P < 0.01 vs. PH-Tau_low_ mice) (Fig. 2C). This indicates that formation of insoluble tau aggregates is PH-Tau dependent. We next examined whether the insoluble tau species aggregated into tau filaments or neurofibrillary tangles, by Thioflavin S or Thiazine Red staining. We found no tangles in any bigenic mice up to 24 months of age (data not shown), indicating a lack of β-sheet structures. Previous studies indicated that the early clusters of both aggregated and less polymerized tau molecules lead to the formation of tau oligomers of different lengths which are still randomly distributed and do not have fully formed β-pleated sheet structures^41^.

### PH-Tau induces cognitive impairment

**Figure 3:**
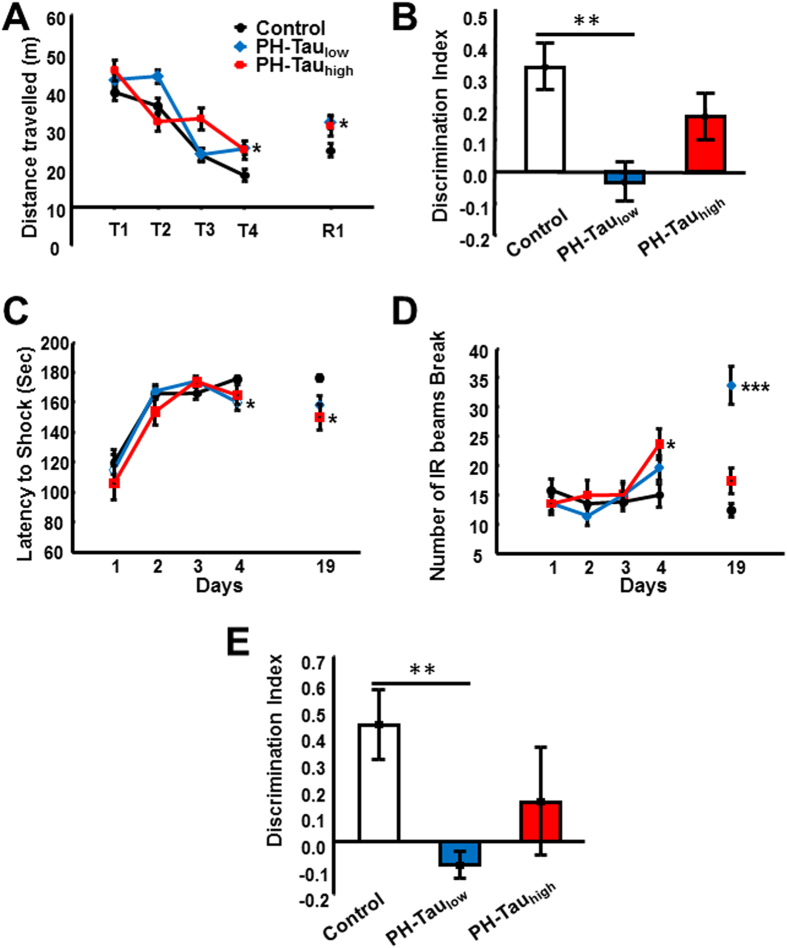
Decreased cognitive behaviors observed in bigenic mice. Bigenic mice (12-month old) were tested for behavior deficits in the (**A**) Morris Water Maze, (**B**,**E**) Novel Object Recognition (12-month old and 5-month old, respectively), and (**C,D**) Passive Avoidance tasks. Significant decreases in spatial memory and memory storage were observed. In the passive avoidance task there was an increase in anxiety. Significant differences are observed between control and PH-Tau_low_ mice (*P < 0.5, **P < 0.01,***P < 0.001).

Impaired memory is a neurological hallmark of AD, including working and spatial memory, recognition memory and associative learning/memory. The behavior of three groups of mice (PH-Tau_high_, PH-Tau_low_, and control) were tested using the Morris Water Maze (MWM), novel object recognition (NOR), and passive avoidance tests. In the MWM, we found that the ability of bigenic mice to locate the platform was delayed significantly at the end of the training phase compared to the non-transgenic mice (Fig. 3A). Furthermore, one month after training, retention (R1) of the spatial memory was correlated to the last day of training in all groups with control groups performing better than bigenic mice. In the NOR, which is a memory task that relies on the innate exploratory behavior of mice and is used to assess memory, PH-Tau_low_ mice were unable to distinguish the novel from the familiar object, with no significant difference in the percentage of time spent investigating both objects, and displayed a discrimination index significantly lower than control mice (Fig. 3B). The PH-Tau_high_ mice averaged more time on the novel object than the familiar object but the trend of the discrimination index was still lower than control mice. Finally, in the passive avoidance test, there was no significant difference in the acquisition phase of the test between the groups as measured by the latency to receive the foot shock (Fig. 3C). In the retention test, both bigenic groups exhibited a significantly (p < 0.01) decreased latency compared to controls. The aversive nature of the passive avoidance test is anxiety promoting. Interestingly, we observed a delayed onset of anxiety, as measured by activity of mice that was more pronounced in bigenic mice compared to controls on both the last day of training and during retention (Fig. 3D).

To further explore the early stage of memory decline, we examined recognition memory in young mice (5 months old, PH-Tau_low_ mice and age-matched littermates) and found that memory in the PH-Tau_low_ mice was also significantly impaired (Fig. 3E). The very early memory deficits described here suggest that the conformational change in tau induced by combination of phosphorylation at key sites (Ser199, Thr212, Thr231, and Ser262) precedes the formation of tau oligomers and causes memory impairments. Taken together these data suggest that the expression of PH-Tau leads to working and spatial memory deficits, disruption of anterograde memory storage, and an increase in anxiety in our transgenic mice.

In all of the memory tests, both bigenic groups performed worse than the control mice, but the bigenic group in which PH-Tau expression was PH-Tau_low_ performed worse than the group in which expression was induced. Up until the time of induction, the PH-Tau_high_ mice were actually PH-Tau_low_ and therefore had a low level of PH-Tau expression. This suggests that the cognitive impairment seen in PH-Tau_low_ mice can be ameliorated by the induction of PH-Tau expression. As PH-Tau is expressed at a higher level, new impairments may be the result of new injuries in the brain. The cognitive impairments resulting from the different levels of expression may have different origins and the mechanisms need to be elucidated.

### Neuronal and synaptic protein loss due to PH-Tau

**Figure 4:**
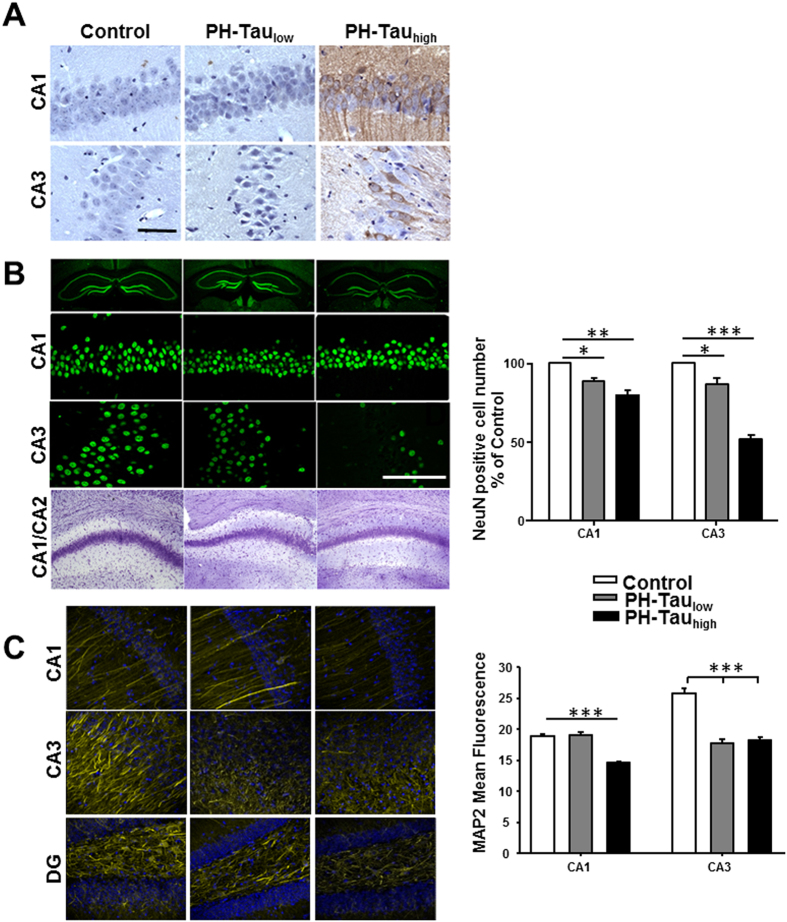
Neuronal loss and synaptic dysfunction in mice expressing PH-Tau. (**A**) Sagittal paraffin sections of hippocampus stained with antibody tau13 recognizing human Tau. The nuclei are counterstained with hematoxylin. (**B**) **Top three panels:** Coronal slices of hippocampus stained with antibody NeuN recognizing nucleus of neurons. **Bottom:** Coronal sections of Nissl staining showed thinner layer of CA1 and CA2 area in bigenic mice compared to control. Scale bar = 50 μm. **Right**: Counts of the NeuN positive cells in both the CA1 and CA3 regions. (**C**) Coronal slices of hippocampus stained with MAP2 shows changes in protein levels in mice expressing PH-Tau. **Right**: Quantitation of the mean fluorescence observed using ImageJ analysis software. (*P < 0.05, **P < 0.01, ***P < 0.001).

**Figure 5:**
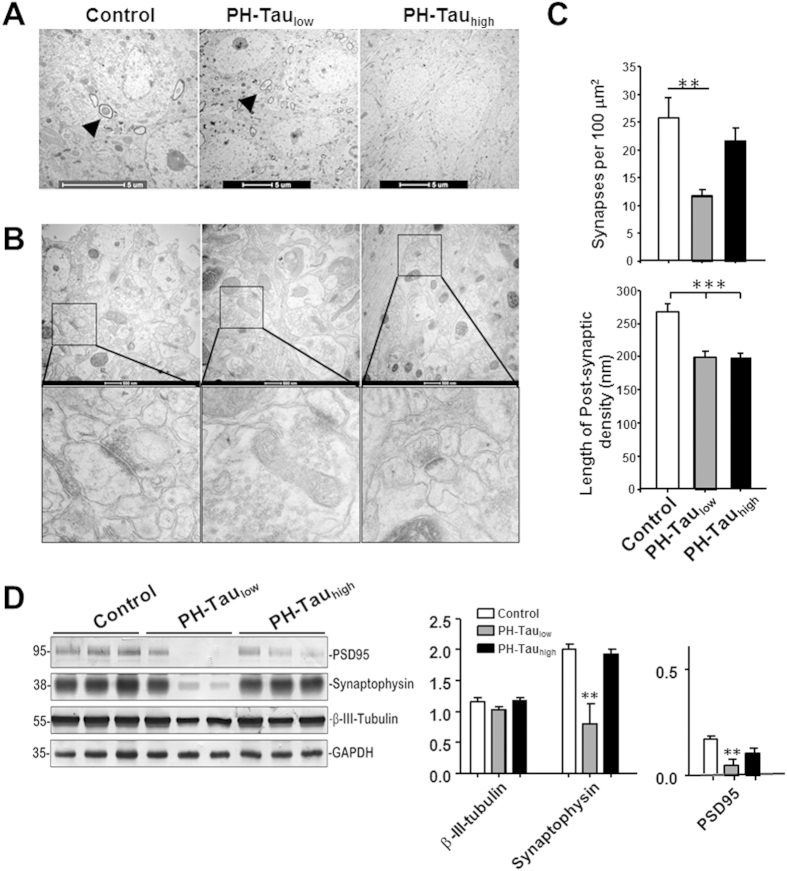
Insufficient developmental dendrite spine and synapse loss in suppressed mice hippocampus. (**A**) CA1 pyramidal neuron cell body layer, myelinated axons are shown in control and PH-Tau_low_ mice (arrow head), but absent in PH-Tau_high_ mice. (**B**) Synapses in CA1 stratum radiatum area, lower panel is the magnified images of square boxes, decreased post-synaptic density and enlarged pre-synaptic portion are shown in PH-Tau_low_ mice. (**C**) Quantitation of the number of synapses in the CA1 stratum radiatum area, significant loss of synapses in PH-Tau_low_ mice is observed. Decrease in the length of the post-synaptic density was observed in both PH-Tau_low_ and PH-Tau_high_ mice. (**D**) Representative Western blot of mouse hippocampus homogenate. The levels of synaptophysin, PSD95, and β-III-tubulin were measured by densitometry and normalized with the levels of GAPDH (*P < 0.05, **P < 0.01, ***P < 0.001).

PH-Tau_high_ mice showed accumulation of PH-Tau in the cell bodies and dendrites of cortical and hippocampal neurons at 18 months of age (Fig. 4A). Less immunoreactivity was observed in hippocampal neurons of PH-Tau_low_ mice and was mostly localized in cell bodies and nuclei, and increased nuclear intensity was observed with the counterstain hematoxylin. Although neuronal loss is an invariable feature of AD, most mouse models do not show neurodegeneration^14^ . To determine whether PH-Tau induces hippocampal neuronal loss, coronal sections from bigenic and non-transgenic mice were stained using neuronal markers. We observed a significant decrease of ~20% of CA1 and ~60% in CA3 NeuN-positive neurons in PH-Tau_high_ mice compared to non-Tg mice (Fig. 4B). Furthermore, CA1/CA2 areas were thinner as shown by Nissl staining (Fig. 4B, bottom). Surprisingly, tau oligomers in PH-Tau_low_ mice also appear to be sufficient for subtle pathological changes, as shown by decreases in NeuN-positive neurons (~11% in CA1, ~14% in CA3) (Fig. 4B). These findings were mirrored in the decrease of processes seen by the staining using the MAP2 antibody in both PH-Tau_low_ and PH-Tau_high_ mice (Fig. 4C). In the CA1 region of the PH-Tau_low_ mice some MAP2 neurites appear to be brighter than those in the control mice. Similarly in the DG region there is a decrease in MAP2 staining of PH-Tau_high_ and PH-Tau_low_ mice with brighter neurites observed in the PH-Tau_low_ mice. These results were enhanced by an observable loss of myelinated axons in the CA1 area of PH-Tau_high_ mice compared to both control and PH-Tau_low_ mice by TEM (Fig. 5A). Collectively, the data indicate that pseudophosphorylation of tau leads to neuronal loss as PH-Tau expression levels increase.

The subtle neurodegeneration seen in PH-Tau_low_ mice seems to be disproportional to the severe memory decline indicating a mechanism different from neuronal loss. To investigate potential mechanisms, we analyzed the expression levels of synaptic proteins and quantitated the number of synapses in CA1 stratum radiatum. PH-Tau_low_ mice showed significant decrease in the number of synapses observed by TEM (Fig. 5B,C). Both PH-Tau_low_ and PH-Tau_high_ mice had a decrease in overall length of post-synaptic densities (Fig. 5B,C). Furthermore, this synaptic disruption was observed biochemically in PH-Tau_low_ mice in the level of synaptic proteins PSD95 (0.050 ± 0.028 vs. control 0.17 ± 0.01, P < 0.01) and synaptophysin (0.80 ± 0.32 vs. control 2.0 ± 0.08, P < 0.01) (Fig. 5D). PH-Tau_high_ mice do not show significant differences from control mice. This loss of synapses suggests that the memory impairment that is observed is mostly correlated with synaptic dysfunction. Furthermore, the oligomeric state of PH-Tau observed in the PH-Tau_low_ mice may play a significant role in change the activity of the synapses. When these oligomers are reduced, as observed in PH-Tau_high_ mice, there is a slight recovery of cognitive function and synaptic proteins indicating that two separate mechanisms might be responsible for cognitive decline: at low levels of PH-Tau there is synaptic dysfunction whereas with higher concentrations there is neuronal death.

### PH-Tau induced astrocytic activation

**Figure 6:**
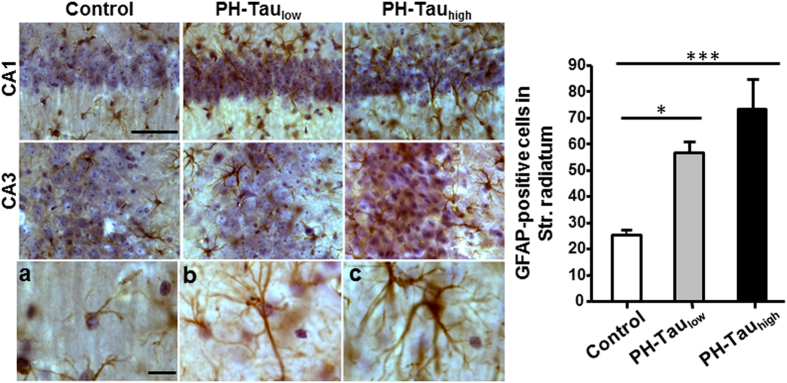
Astrocytosis of bigenic mice. **Left**: Coronal sections stained with GFAP antibody showed activation of astrocytes in CA1 and CA3 area of bigenic mice brains, with recognized morphological changes, including enlarged size and numerous cytoplasmic processes (**b,c**). **Right**: The numbers of CA1 stratum radiatum (str. radiatum) GFAP positive cells were analyzed by unbiased stereological estimates (*P < 0.05). Scale bar = 50 μm.

Astrocytic activation characterized by the upregulation of glial fibrillary acidic protein (GFAP), cell proliferative and morphological alterations occur in response to neuronal damage or can induce damage to neuronal structures^42^. The relationship between neuronal loss and astrocytic activation was examined in the CA1 stratum radiatum astrocytes by unbiased stereological observation. The number of GFAP positive cells in PH-Tau_low_ mice (56.5 ± 4.5) was increased significantly compared to control mice (25.2 ± 1.9, P < 0.05), and increased further in PH-Tau_high_ mice (73 ± 11, P < 0.001) (Fig. 6). Moreover, many astrocytes ectopically invaded the CA1 pyramidal layer in both bigenic mice, resulting in morphological changes, including enlarged size and numerous cytoplasmic processes.

## Discussion

In the present study, we report a new inducible tauopathy mouse model in which pseudophophorylated tau is expressed in neurons of the mouse forebrain regulated by doxycycline in the diet. In the PH-Tau_high_ mice, with a PH-Tau expression of about 14% of the endogenous tau, a significant increase in memory deficits was observed when compared to the controls. The cognitive impairments appear to be a result of progressive neuronal loss as determined by decreased immunoreactivity to neuronal markers such as NeuN and MAP2 and the loss of myelinated axons in CA1, probably coming from the CA3 and entorhinal cortex. Unexpectedly, we found significant memory deficits in PH-Tau_low_ bigenic mice. These animals are leaky for PH-Tau expression giving basal levels up to 4% of total tau. Neuronal loss in these animals was not as pronounced as in PH-Tau_high_ mice, nevertheless PH-Tau expression was accompanied by severe memory deficits, synaptic loss and condensation in neuronal nuclei. Taken together, these results suggest that in our model the cognitive impairment could be triggered by two different mechanisms: at low level PH-Tau expression induced synaptic dysfunction and at higher concentration PH-Tau induced neuronal loss.

When normal tau is expressed in N2A cells there is an induction of neurite extension. Instead, when PH-tau is expressed, no neurites are formed and PH-Tau is translocated in the nucleus. In our bigenic mice fed with doxycycline, basal PH-Tau expression was detected (4% of the endogenous protein). Interestingly, in immunohistochemistry only the neuronal nuclei were slightly positive for human tau. Tau has been shown to be present in the nucleus of the neurons at least 20 years ago^43^. The functions of tau in the nucleus of neurons are unclear, and it is a very exciting fact that is very much investigated. For example, nuclear tau has been proposed to protect neuronal DNA from stress^44^, to play a role in Huntington disease^45^, and to participate in neurodegeneration by controlling chromosome relaxation^46^, to cite some of the current and exciting literature.

When PH-Tau is expressed at low levels, it is conceivable that the concentration is not enough to induce large aggregates and PH-Tau can translocate into the nucleus as it was shown in cells^29^ or in different disease situation^45^. In the nucleus, PH-Tau may exert a toxicity that might involve an unknown nuclear function of tau, like the heterochromatin relaxation recently described^46^ or to interact with different proteins, perhaps membrane proteins, in a pore like structure as seen in PSP^47^. In the present report, we could not detect the presence of PH-Tau by conventional SDS-PAGE analysis so we needed to modify the protocol, suggesting that under these conditions it is engaged in an interaction with other protein/proteins. We detected it as an oligomer, as it has been previously described for tau^39^. Tau-induced early cognitive impairment might recapitulate the 10-fold increase in the probability of getting AD for adults that performed poorly in memory tests in the absence of any obvious symptoms of the disease^48^. The bigenic mouse model will be useful to study early detection of the disease and the slow progressive deterioration of other tauopathies, like Chronic Traumatic Encephalopathy^3^,^49^,^50^.

As PH-Tau concentration increases, aggregates might be formed in the cytoplasm preventing tau translocation in the nucleus. PH-Tau might be interacting under these conditions with the normal murine tau and maybe other MAPs, disrupting microtubules, known to be decrease in AD^49^. In our model, we can detect PH-Tau in the sarkosyl insoluble fraction, supporting this scenario, and a significant decrease in the myelinated axons in CA1. This neurodegeneration process might trigger apoptosis. We detected neuronal loss. Interestingly, the synaptic dysfunction recovers when the PH-Tau concentration increases, both by the level of synaptic proteins and by electron microscopy, arguing strongly that the cognitive impairment in the animals with higher levels of PH-Tau is different from the one observed in PH-Tau_low_ animals. In low PH-Tau levels, the effect could be at a nuclear level, with changes in protein expression, whereas at higher PH-Tau level, the cognitive impairment could be the result of neuronal death.

Upon induction, PH-Tau appeared mostly to be truncated at D421. This site on tau has been reported to be cleaved by caspase-3 early in disease progression, suggesting that the increase in PH-Tau induces caspase-3 activation starting a process of neuronal death via apoptosis^36^,^51^. DeCalignon *et al.*^52^, using live-imaging, proposed that caspase activation precedes and leads to tangle formation. In our model, we detect the products of caspase activation, suggesting that tau phosphorylation precedes caspase activation. We did not detect tangle formation in our model, perhaps because the concentration of PH-tau is lower than that expressed in other models. Our data are in agreement with the AD pathology studies, which show a well-defined pathway with phosphorylation as the earliest event when compared with other pathological events such as cleavage at site D421 and the canonical Alz-50 conformational change according to Braak stages II–V^41^,^53^,^54^,^55^.

Further evaluation indicated that the brains of PH-Tau_high_ mice have high levels of astrocytosis indicating inflammation. The findings in this study strongly indicate that the progression of activated astroglia is directly related to neuronal loss as the expression of PH-Tau increases. In agreement with our results, studies in human brains have found a linear increase in astrocytosis and microgliosis together with increases in NFT formation and disease evolution^56^. In addition, astrocytosis was determined to be an early phenomenon before amyloid deposits in AD development. Although the prevailing view is that astrocytes respond secondarily to neuronal damage, there is abundant and growing evidence supporting the role of these cells as active participants in many of the mechanisms that are associated with the pathophysiology of AD. Interactions between dysfunctional astrocytes and neighboring neurons can initiate a cascade of events that culminates in neuronal injury and the expression of hallmark lesions of AD.

Taken together, the PH-Tau mouse model that we have generated appears to result in cognitive deficits that are driven by two separate mechanisms. In early stages, where expression of PH-Tau is low, there is synaptic dysfunction which is indicated by a loss of synapses and condensation of neuronal nuclei. There is some neuronal loss, but this does not appear to be the main cause of the behavioral deficits. When the expression of PH-Tau is induced, correlating to later stages where PH-Tau becomes sarkosyl-insoluble, there is highly significant neuronal loss and astrocytosis in the hippocampus even as observed cognitive deficits are apparently alleviated and synaptic proteins are recovered. This neuronal loss may be linked to a breakdown of the microtubules leading to apoptotic cell death as has been seen in culture^29^,^30^ and suggested by PH-Tau truncation at D421, a caspase-3 cleavage site on tau^29^,^36^,^57^.

Using this inducible mouse model, we show that substoichiometric amounts of pathological tau are able to seed formation of oligomers, impair synaptic functions, and induce neurodegeneration and early onset of cognitive impairments. To our knowledge, it is the first time that a form of tau expressed at such low levels can induce these marked effects, strongly arguing that this is the toxic tau-species in neurodegeneration. We produced a specific mouse model that could be used to account for the molecular and functional diversity that is involved in the astrocyte dysfunction that is associated with the development and progression of AD. Finding an effective therapy for AD-type tauopathies is a major unmet medical need. This model could also help to identify a potential target for future treatment of AD.

## Materials and Methods

### Animals

All procedures involving mice were approved by the Institutional Animal Care and Use Committee of the College of Staten Island. The activator mouse line (CaMKIIα-*tTA* mice) in which the *tTA* transgene is under control of the CaMKIIα promoter was purchased from The Jackson Laboratories. Responder mice were generated using the transcription unit encoding the human longest Tau isoform with S199E, T212E, T231E, S262E, and R406W^29^ cloned into the pTRE-Tight-BI-ZsGreen1 vector (Clontech Laboratories) using the In-Fusion Kit following the manufacturer’s instructions. The DNA microinjection was performed by The Jackson Mice and Services Laboratories in C57BL/67 strain. Mice harboring responder or activator transgenes were bred to generate bigenic progeny containing both transgenes. Expression of PH-Tau was induced by withdrawal of doxycycline, and suppressed by addition of doxycycline (1 g/kg in the food, BioServ Inc) (Tet-Off system). Protocols involving animals were approved by the College of Staten Island (CSI) Human & Animal Research Protection Program Office in accordance with the Guide for the Care and Use of Laboratory Animals and CSI’s Office of Laboratory Animal Welfare approved Public Health Service Animal Welfare Assurance, #A3718-01. All efforts were made to reduce animal numbers used to the minimum required for valid statistical analysis.

### Cell culture and transfection

Mouse neuroblastoma cells (Neuro-2A or N2A) was a kindly gift of Dr. Fei Liu (NY State Institute for Basic Research). For transfection, approximately 1 × 10^5^ cells per well were plated into 12-well plate, and were roughly 80% confluent in 24 hrs. N2A cells were transiently transfected with normal and PH-Tau with Lipofectamine 2000 (Invitrogen) according to manufacturer instructions. Differential Interference Contrast (DIC) and fluorescence images were taken after 24 hours of transfection. Images obtained by confocal microscopy were used to determine nuclear localization. Nuclear regions from at least 75 cells were analyzed for green fluorescence using ImageJ. The mean fluorescence was average for each group and compared.

### Tissue extraction and Western blotting

For Western blotting analysis with or without fractionation, mice brain tissue was lysed using RAB buffer as described previously^58^ (for details see SI). The tissue was ground using a tissue grinder and the resulting homogenates were sonicated and either boiled for 5 min or incubated at 37 °C for 1 h in 2X SDS-PAGE loading buffer. The homogenates were used for SDS-PAGE and Western blot analysis. The levels and solubility of tau were determined by sarkosyl extraction^58^. The amount of homogenates and sarkosyl-insoluble pellet represented ~0.7% and 1.2% of the total material, respectively. For quantification of tau levels, the western blots were probed with pan-tau antibody DA9 and analyzed by densitometry.

### Immunohistochemistry and Immunofluorescence

Transgenic and control mice from 15–24 months were anesthetized and transcardially perfused sequentially with 0.1 M phosphate buffered saline (PBS) and 4% paraformaldehyde in 0.1 M PBS or 1% paraformaldehyde and 1% glutaraldehyde in 0.1 M PBS (pH 7.4)^59^. Brains were removed and further fixed by immersion with the same solution above at 4 °C for 1 week. Some samples were embedded in paraffin and cut sagittally on a sliding microtome at a thickness of 6 μm. Cryosections in the coronal plane (40 μm) were cut on a cryostat and stored at −20 °C in a solution with 30% Ethylene Glycol and Sucrose in 0.1 M PBS. Coronal vibratome sections (50 μm) were cut and stored at 4 °C in 0.1 M PBS.

Paraffin sections were deparaffinized, rehydrated, and washed and endogenous peroxidase was quenched. Epitope retrieval was done dependent on the primary antibodies and performed in citrate buffer for 1 min in microwave. After blocking in 10% normal goat serum for 1 h, primary antibodies were incubated overnight at 4 °C in the present of 2% BSA and normal goat serum. HRP-conjugated secondary antibodies were incubated at room temperature for 30 min. All washing steps and antibody dilution were done using 0.01 M PBS (pH 8.0) or Tris-buffered saline (TBS). Incubation and detection with SignalStain DAB Substrate Kit (Cell Signaling) were done according to the manufacturer’s manual. Some sections were counterstained with Hematoxylin.

For immunofluorescence, a PBS-0.2% Triton X-100 (PBST)(Sigma Chemical. Co., St. Louis, MO) solution was used in all washing steps. Free floating sections were placed in wells of 24-well plates and were rinsed for 10 min in PBST and blocked for 60 min with blocking buffer (BB). Slices were then incubated overnight at 4 °C under slight agitation with primary antibody dissolved in BB. Next day, slices were incubated for 2 h at room temperature in the dark with secondary antibody diluted in BB. After washings, slices were incubated with Topro III for 25 min, after extensive washings slices were mounted onto gelatin-coated slides using mounting medium.

Slices were observed under a LEICA TGS SP5 confocal laser scaning microscope (Leica Microsystems CMS GmbH, Mannheim, Germany). All stereological cell counts were performed blind to genotype. Three sections per animal were sampled and all measurements were repeated three times.

### Antibodies

See Supplemental Information for antibody information.

### Nissl, Thioflavine-S and Thiazin red staining

Coronal sections were collected in order, mounted on slides, dried overnight at room temperature, stained with cresyl violet, dehydrated, and coverslipped. For thioflavine-S staining, the free-floating sections stained in 0.1% thioflavine-S according to manufacturer’s protocol (Sigma) mounted in 80% glycerol at pH 4.0. Thiazin red (TR) staining was performed as previously described^60^ using aqueous 0.001% TR (gift from Dr. Jose Luna-Munoz).

### Electron Microscopy

Mice were perfused with 4% glutaraldehyde in 0.1 M sodium cacodylate buffer and brains were postfixed overnight and rinsed. Coronal vibrotome sections at 200 were treated with 1% osmium tetroxide, 1.5% potassium ferracyanide in 0.1 M cacodylate buffer, dehydrated in ethanol solutions, stained en bloc in 3% uranyl acetate in 70% ethanol, and then further dehydrated in ethanol followed by propylene oxide. Tissue was infiltrated in resin (Embed 812 kit; Electron Microscopy Sciences) and then embedded. The CA1 sections were cut, and random images were acquired through the stratum radiatum. At least ten images were analyzed per sample. ImageJ was used to count the number of synapses and measure the length of the post-synaptic density.

### Memory and Learning

Mice ranging in age from 15–24 months were subjected to the Novel Object Recognition^61^, Morris Water Maze^62^ and Passive Avoidance^63^ tasks. The details for these trials are in Supplemental Information.

### Statistical Analysis

All data are presented as average ± SEM. Statistical analysis was performed using STATISTICA software. N = 3–11 mice/group were used for each experiment. P-values less than or equal to 0.05 were considered statistically significant.

## Additional Information

**How to cite this article**: Di, J. *et al.* Abnormal tau induces cognitive impairment through two different mechanisms: synaptic dysfunction and neuronal loss. *Sci. Rep.* **6**, 20833; doi: 10.1038/srep20833 (2016).

## Supplementary Material

Supplementary Information
