# Supplementary material for: Abnormal tau induces cognitive impairment through two different mechanisms: synaptic dysfunction and neuronal loss
Source: Sci Rep. 2016 Feb 18;6:20833. doi: 10.1038/srep20833 (PMC4757872; doi:10.1038/srep20833)
Supplement: Supplementary Information [file srep20833-s1.pdf]

Abnormal tau induces cognitive impairment through two different mechanisms: synaptic dysfunction and neuronal loss

Running Title: Tau induced cognitive impairment through two mechanisms

J. Di[1‖], L.S. Cohen[1‖], C. P. Corbo[2], G.R. Phillips[1], A. El Idrissi[1], A.D. Alonso[1*]

[1]Department of Biology and Center for Developmental Neuroscience, College of Staten Island, City University of New York, Staten Island, NY 10314, USA

[2]Department of Biological Sciences, Wagner College, Staten Island, NY 10301, USA

[*]These authors contributed equally to the manuscript.

[‖]These authors contributed equally to this work.

[*]To whom correspondence should be addressed. Alejandra Alonso, Department of Biology and Center for Developmental Neuroscience, College of Staten Island, City University of New York, Staten Island, NY 10314, USA. (718) 982-4153. Alejandra.Alonso@csi.cuny.edu

Supplemental Information

Transgenic Constructs & Animals. All procedures involving mice were carried out in accordance with the guidelines approved by the Institutional Animal Care and Use Committee of the College of Staten Island. The activator mouse line (CaMKIIα-*tTA* mice) in which the *tTA* transgene is under control of the CaMKIIα promoter was purchased from The Jackson Laboratories. Responder mice were generated using the transcription unit encoding the human longest Tau isoform with S199E, T212E, T231E, S262E, and R406W was generated previously [1]. To clone into the pTRE-Tight-BI-ZsGreen1 vector (Clontech Laboratories), the In-Fusion Kit

was used following the manufacturer's instructions. Primers designed for PCR amplification were infusion_1 (5'-CGGTACCCGGGGATCCATGGCTGAGCCCCGCCAGGA-3') and infusion_2 (5'-GCTGACTAGAGGATCCTCACAAACCCTGCTTGGCCAGGGA-GG3'). The vector was linearized with BamHI prior to the In-Fusion reaction. Resulting clones were sequenced for verification and then linearized with TaqII (Roboklon, Denmark).The DNA microinjection was performed by The Jackson Mice and Services Laboratories in C57BL/67 strain. Mice harboring responder or activator transgenes were bred to generate bigenic progeny containing both transgenes. The expression can be switched on by withdrawal of doxycycline, and switched off by addition of doxycycline (1g/kg in the food, BioServ Inc) (Tet-Off system). The offspring of mice were screened by PCR using the primer pair 5'-CGCTGTGGGGCATTTTACTTTAC-3', 5'-CATGTCCAGATCGAAATCGTC-3' for *tTA*, and 5'-GCTCGTTTAGTGAACCGTCAG-3', 5'-CGTGTCACCCTCTTGGTCTT-3' for TRE. The littermate wild-type mice were used as controls. Both males and females were used. Protocols involving animals were approved by the College of Staten Island Human & Animal Research Protection Program Office in accordance with the Guide for the Care and Use of Laboratory Animals and CSI's Office of Laboratory Animal Welfare (OLAW) approved Public Health Service (PHS) Animal Welfare Assurance, #A3718-01. All efforts were made to reduce animal numbers used to the minimum required for valid statistical analysis.

Cell culture and transfection. Mouse neuroblastoma cells (Neuro-2A or N2A) were a kindly gift of Dr. Fei Liu (NY State Institute for Basic Research). The cells were kept in 10% FBS DMEM (high glucose, L-gluatmine) medium in 5% CO_2/37 °C. For transfection, approximately $1x10^5$ cells per well were plated into 12-well plates, and were roughly 80% confluent in 24 hrs. Wild-type human 2N4R tau or PH-tau DNA constructs (1 µg) were added to 100 µl of OptiMEM. Lipofectamine 2000 (Invitrogen) (2 µl) was added to a new 100 µl of OptiMEM, incubated for 5 min, and mixed with the DNA mixture then incubated 20 min at room temperature. The growth medium was removed from cell cultures, rinsed once with 1 ml OptiMEM, replaced with another

1 ml OptiMEM. The preincubated DNA/Lipofectamine solution were added to the cells, mixed gently and incubated in 5% CO_2/37 °C incubator for 4-6 hrs. The OptiMEM was replaced with DMEM growth medium and cultured for another 16 hrs. The cells were observed and imaged with a Zeiss Axio Observer at 5% CO_2/37 °C. Both Differential Interference Contrast (DIC) and fluorescence images were taken. . Images obtained by confocal microscopy were used to determine nuclear localization. Nuclear regions from at least 75 cells were analyzed for green fluorescence using ImageJ. The mean fluorescence was average for each group and compared.

Tissue extraction and Western blotting. For Westerns without fractionation, hippocampal tissues were lysed by RAB buffer (100 mM Mes, 1 mM EGTA, 0.5 mM $MgSO_4$, 750 mM NaCl, and 20 mM NaF) containing protease inhibitors with 30 strokes using a tissue grinder [2]. The homogenates were sonicated for 6 pulses at 50% duty cycle, incubated at 4°C for 30min, after mixed with 2x SDS-PAGE loading buffer, either boiled for 5min or incubated at 37°C for 1h. The homogenates were used for SDS-PAGE, transferred to PVDF membranes and deteted using the ECL method (Pierce). Protein levels were quantified using ImageJ (National Institute of Health).

Preparation of soluble and insoluble fractions was performed as described with some modifications[2]. The forebrains were homogenized in 10 volumes, 300 μl of the homogenates were centrifuged at 14,000 rpm for 20 min and the supernatants were collected as RAB fraction. The pellets were resuspended with 500 μl of RIPA buffer (50 mM Tris-HCl at pH 8.0, 150 mM NaCl, 1% Nonidet P-40, 5 mM EDTA, 0.5% sodium deoxycholate, and 0.1% SDS) containing protease inhibitors, sonicated briefly and then incubated on ice for 30 min. The homogenates were centrifuged at 14,000 rpm for 20 min and the supernatants were collected as RIPA fraction. The pellets were resuspended with 500 μl of RAB buffer containing 1% N-lauroylsarcosine (sarkosyl) in Mes buffer (20 mM Mes at pH 6.8, 80 mM NaCl, 1 mM $MgCl_2$, 2 mM EGTA, 10 mM NaH_2PO_4, and 20 mM NaF) with protease inhibitors, vortexed for 30 min at

15~25 °C, incubated overnight at 4 °C, and then centrifuged at 14,000 rpm for 30 min at 15~ 25 °C. The pellets (sarkosyl-insoluble fraction) were resuspended in 100 μl of 2X protein loading buffer and incubated in a boiling water bath for 5 min. All the above procedures prior to boiling in SDS-sample loading buffer were carried out on ice. Homogenates and insoluble fractions were loaded per gel for SDS-PAGE and Western blot analysis.

Immunohistochemistry and Immunofluorescence. Transgenic and control mice from 15-24 months were anesthetized and transcardially perfused sequentially with 0.1M phosphate buffered saline (PBS) and 4% paraformaldehyde in 0.1M PBS or 1% paraformaldehyde and 1% glutaraldehyde in 0.1M PBS (pH 7.4) [3]. Brains were removed and further fixed by immersion with the same solution above at 4°C for 1 week. Some samples were embedded in paraffin and cut sagittally on a sliding microtome at a thickness of 6 μm. Cryosections in the coronal plane (40 μm) were cut on a cryostat and stored at -20°C in a solution with 30% Ethylene Glycol and Sucrose in 0.1M PBS. Coronal vibratome sections (50 μm) were cut and stored at 4°C in 0.1M PBS.

Paraffin sections were deparaffinized, rehydrated, and washed. Endogenous peroxidase was quenched by treating the section with methanol containing 0.3% H_2O_2 for 30 min. Epitope retrieval was done dependent on the primary antibodies and performed in citrate buffer (1.8 mM citric acid, 8.2 mM sodium citrate, pH 6.0) for 1 min in microwave. After blocking in 10% normal goat serum for 1 h, primary antibodies were incubated overnight at 4°C in the present of 2% BSA and normal goat serum. HRP- conjugated secondary antibodies (anti-mouse IgG, IgM, and anti-rabbit IgG, Cell Signaling) were incubated at room temperature for 30 min. All washing steps (3×15 min) and antibody dilution were done using 0.01M PBS (pH 8.0) or *Tris-buffered saline* (*TBS*, 50 mM Tris-Cl, 150 mM NaCl, pH 7.5). Incubation and detection with SignalStain DAB Substrate Kit (Cell Signaling) were done according to the manufacturer's manual. Some sections were counterstained with Hematoxylin.

For immunofluorescence, a PBS-0.2% Triton X-100 (PBST)(Sigma Chemical. Co., St. Louis, MO) solution was used in all washing steps. Free floating sections were placed in wells of 24-well plates and were rinsed for 10 min in PBST and blocked for 60 min with blocking buffer (BB) containing 10% Normal Goat Serum-10% Normal Horse Serum in PBST. Slices were then incubated overnight at 4°C under slight agitation with primary antibody dissolved in BB. Next day, slices were incubated for 2h at room temperature in the dark with AlexaFluor 488 conjugated donkey anti-rabbit or mouse IgG (1:500, Invitrogen Life Sciences, Carlsbad, CA, USA) secondary antibody diluted in BB. After washings, slices were incubated with 0.001% Topro III dissolved in PBST for 25 min, after extensive washings slices were mounted onto gelatin-coated slides using Vectashield hard set with DAPI (Vector Laboratories) as a mounting medium.

Slices were observed under a LEICA TGS SP5 confocal laser scanning microscope (Leica Microsystems CMS GmbH, Mannheim, Germany). Confocal scans were taken at 2 μm z-steps keeping all parameters (pinhole, contrast and brightness) constant. Image analyses were conducted on image z-stacks which contained the field of interest. Images were assembled into montages using Adobe Photoshop (Adobe Systems, Mountain View, CA, USA). Immunohistochemical staining controls were performed by omitting the primary or secondary antibodies to confirm the specificity of the staining.

The coronal sections of the dorsal hippocampus at antero-posterior positions from bregma between-1.34mm and -2.3mm were selected for NeuN-(neurons) or GFAP-positive cells (astrocytes) estimates. The regions sampled included the CA3 and CA1 regions of the hippocampus for neuronal and CA1 stratum radiatum for astrocyte populations. All stereological cell counts were performed blind to genotype. Three sections per animal were sampled and all measurements were repeated three times.

Antibodies. The following primary antibodies were used: mouse monoclonal antibodies tau13 (human tau 9-18, 1:20000 for immunostaining, 1:100000 for western blot; gift from Dr. L.

Binder), 499 (human tau N-terminus, 1:200; gift from Dr. P.Davies), DA9 (human and mouse tau 102-140, 1:100; gift from Dr. P. Davies), tauC3 (truncated tau at Asp421, 1:1000; gift from Dr. L. Binder), tau46 (tau 404-441, 1:1000; Upstate); a rabbit polyclonal anti-neuronal nuclei (NeuN, 1:400; Cell Signaling Technology, Inc., Danvers, MA, USA) for neurons; a rabbit polyclonal anti-glial fibrillary acidic protein (GFAP, 1:500; Millipore AB5840) for astrocytes; a chicken polyclonal anti-MAP2 (1:500, Abcam); Synaptophysin (clone SY38, 1:500; Millipore); PSD95 (D27E11, 1:1000; Cell Signaling); GAPDH (GA1R, 1:5000; Fisher). The following secondary antibodies were used: donkey anti-mouse IgG HRP(1:5000;Thermo); goat-anti-rabbit IgG HRP(1:2000; Millipore); Alexa Fluor488 goat-anti-mouse IgG (1:500; Molecular Probes); Alexa Fluor488 goat-anti-rabbit IgG (1:500; Molecular Probes); Alexa Fluor555 donkey-anti-chicken IgG (1:500, Molecular Probes)

Nissl, Thioflavine-S and Thiazin red staining. Coronal sections were collected in order, mounted on slides, dried overnight at room temperature, stained with cresyl violet, dehydrated, and coverslipped. For thioflavine-S staining, the free-floating vebratome sections were washed in distilled water for 1 min and then incubated for 10 min in 0.1% thioflavine-S (Sigma) made in distilled water, then dipped in 70% ethanol two times and incubated in 100mM sodium citrated, pH 4.0, for 4 min, mounted on slides, and coverslipped in 80% glycerol at pH 4.0. For Thiazin red(TR) staining, after washing, the sections were incubated for 15 min in aqueous 0.001% TR(gift from Dr. Jose Luna-Munoz), rinsed with water, then mounted and coverslipped with Vectashield hard set with DAPI (Vector Laboratories) [4].

Electron Microscopy. Mice were perfused with 4% glutaraldehyde in 0.1 M sodium cacodylate buffer and brains were postfixed overnight and rinsed. Coronal vibrotome sections at 200 μm were cut and the hippocampi were dissected out. Tissue was treated with 1% osmium tetroxide, 1.5% potassium ferracyanide in 0.1 M cacodylate buffer for 1 h at 4 °C and then dehydrated in ethanol solutions (50%, 60%, and 70%). Tissue was stained en bloc in 3% uranyl acetate in 70% ethanol for 12 h at 4 °C and then further dehydrated in 80%, 90%, and 100% ethanol

followed by propylene oxide. Tissue was infiltrated with a 1:1 solution of resin (Embed 812 kit; Electron Microscopy Sciences) and propylene oxide for 24 h at room temperature followed by pure resin for several hours. Hippocampi were then flat embedded in the cap of a BEEM capsule.

Area CA1 (including cell bodies and stratum radiatum) was trimmed on the block face and serial sections cut and mounted on Formvar-coated slot grids. Sections were contrasted with uranyl acetate and lead citrate and visualized on a Fei Tecnai Spirit transmission electron microscope at 80 keV. For synapse analysis, random images were acquired through the stratum radiatum at 18,000 x magnification. The CA1 sections were cut, and random images were acquired through the stratum radiatum. At least ten images were analyzed per sample. ImageJ was used to count the number of synapses and measure the length of the post-synaptic density.

Object Recognition Task. Mice at 18-24 months (n=7 or 8 per group) or 5 months (n=3) were tested in an open-square white arena, 60*60cm, 40cm high as previously described [5]. The following objects were used: two black metal cylinders, 6*7cm; an orange disk, 1.5*5cm. The task started with a habituation trial, during which the animals were placed in the empty arena for 10 min. The next day, mice were again place in the same arena containing two identical objects (familiarization phase). Exploration was recorded in a 10-min trial. Sniffing, touching, and stretching the head toward the object at a distance of no more than 2cm were scored as object investigation. Six hours later (test phase), mice were again placed in the arena containing two objects: one identical to one of the objects presented during the familiarization phase (familiar object), and a new, different object (novel object). The time spent exploring the two objects was recorded for 10 min. Memory was expressed as a discrimination index, namely (seconds on novel-seconds on familiar)/(seconds on novel+seconds on familiar) and was expressed as the percentage of time on each object.

Morris Water Maze. Fifteen-month old mice were trained during a 4 day training period and were tested in four trials per day for their ability to locate a hidden platform aided by visual clues

surrounding the water pool as previously described [6]. Mice were allowed free access to food and water and maintained at constant temperature (25°C). Spatial memory was measured by Morris water maze (142 cm in diameter, opaque water, 26±1°C, automated swim-path monitoring; HVS Image). Mice were subjected to a training period for 4 days and were then tested a month later for their ability to search for the platform in the same water pool with visual clues, but where the platform had been removed. Hidden platform training is a total of 20 trials/day, 11×11×39 cm hidden platform, placed 30 cm from the wall and 2 cm bellow the water level). At the end of the training, a 60-s probe trial (platform absent) was administered. Through these training sessions, mice acquired spatial memory about location of the safe platform. The time mice stay in the previous platform quadrant (quadrant time) and their swimming path were recorded by a video camera. Videos were subsequently analyzed by AmyMaze software. The longer a mouse stays in the previous platform-located quadrant, the better it scores spatial memory. The spatial memory of a mouse was expressed as quadrant time (%).

Passive avoidance task. Using passive avoidance paradigm, we tested the ability of mice to learn to avoid an electrical shock as previously described [7]. Mice received six trials a day for five days and measured their learning after repetitive training. The apparatus has a bright and a dark compartment with a computer-controlled door between them. The delivery of electric shocks and the raising and lowering of the door and the latencies at which the animals stepped into the dark from the bright compartment were controlled by the computer. Each animal was gently placed in the light compartment for 10 s, after which the guillotine door was raised and the time the animal waited before crossing to the dark (shock) compartment was recorded as the latency. The trial ended when an animal waited more than 180s to cross to the other side, or if it received an electrical shock in the dark side after crossing. Once the animal crossed with all four paws to the next compartment, the door was closed and a 1.5 mA foot shock was delivered for 5 s. Mice that showed immobility from the previous experiment were excluded from this test.

The chambers were cleaned carefully between animals with a tap water solution containing ethanol (5% v/v).

Training sessions: each mouse was trained by gently placing it in the light compartment then when it stepped through the dark compartment putting the 4 paws on the grid floor, the door automatically closed and an electric shock of 0.5 mA was delivered for 3s. Repeat the training 6 times per day, with an upper cut-off time of 180s.

Retention session: each mouse was introduced to the light compartment and the latency to step-through to the dark compartment was recorded as a passive avoidance behavior indicating memory retention, with an upper cut-off time of 180s. No electric shock was delivered during retention session.

Statistical Analysis. All data are presented as average ± SEM. Statistical analysis was performed using STATISTICA software. N = 3-11 mice/group were used for each experiment. P-values less than or equal to 0.05 were considered statistically significant.

References

1. Alonso AD*, et al.* Phosphorylation of tau at Thr212, Thr231, and Ser262 combined causes neurodegeneration. *J Biol Chem* **285**, 30851-30860 (2010).
2. Chun W, Johnson GV. Activation of glycogen synthase kinase 3beta promotes the intermolecular association of tau. The use of fluorescence resonance energy transfer microscopy. *J Biol Chem* **282**, 23410-23417 (2007).
3. Sarsoza F*, et al.* A fibril-specific, conformation-dependent antibody recognizes a subset of Abeta plaques in Alzheimer disease, Down syndrome and Tg2576 transgenic mouse brain. *Acta Neuropathol* **118**, 505-517 (2009).
4. Luna-Munoz J, *et al*. Thiazin red as a neuropathological tool for the rapid diagnosis of Alzheimer's disease in tissue imprints. *Acta Neuropathol* **116**, 507-515 (2008).
5. Antunes M, Biala G. The novel object recognition memory: neurobiology, test procedure, and its modifications. *Cogn Process* **13**, 93-110 (2012).
6. Dobkin C,*et al*. Fmr1 knockout mouse has a distinctive strain-specific learning impairment. *Neuroscience* **100**, 423-429 (2000).
7. El Idrissi A. Taurine improves learning and retention in aged mice. *Neurosci Lett* **436**, 19-22 (2008).
